# Productivity of tasks performance in children and adolescents with different level of media multitasking

**DOI:** 10.1192/j.eurpsy.2021.551

**Published:** 2021-08-13

**Authors:** A. Koshevaya, G. Soldatova, S. Chigarkova

**Affiliations:** Faculty Of Psychology, Lomonosov Moscow State University, Moscow, Russian Federation

**Keywords:** media multitasking, productivity, Children, adolescents

## Abstract

**Introduction:**

Media multitasking (MMT) begins to play an increasingly important role in terms of the digitalization of everyday life. At the same time, the effect of MMT on efficiency is still poorly highlighted, especially among the younger generation (May, Elder, 2018; Patterson, 2017; Peifer, Zipp, 2019).

**Objectives:**

The aim is to identify types of MMT in children and compare them by productivity and time of task performance.

**Methods:**

Quasi-experimental research, which included the performance of tasks on a computer and a smartphone, was conducted among children in three groups aged 7-10 years, 11-13 years, 14-16 years (N=154).

**Results:**

Based on a number of criteria, several types of behavior are identified in the MMT environment: two subgroups of “single-taskers” (23%), “light” (19%), “medium” (54%) and “heavy” (4%) MMT. Comparative analysis (p=0.027) reveals high scores for proper task performance of the subgroup “single-taskers1”, as well as “heavy” and “light” MMT. The other subgroup “single-taskers2” and “medium” MMT show, on the contrary, low productivity results. MMT groups also differ in terms of task performance time (p=0.006). The “light” and “heavy” MMT cope the fastest. The second place by the speed of task performance is held by “medium” MMTs and “single-taskers1”, and the slowest are “single-taskers2”.

**Conclusions:**

The results show that both linear and nonlinear behavioral strategies can lead to a certain level of performance. Most children try to operate in the MMT mode and it is becoming the dominant and ubiquitous modus vivendi for the younger generation. The reported study was funded by RFBR, project No. 19-29-14181.

**Conflict of interest:**

The reported study was funded by RFBR, project No. 19-29-14181.

